# Novel evidence for the nucleocytoplasmic shuttling of HIV-1 Tat by Importin13

**DOI:** 10.1186/1742-4690-10-S1-P102

**Published:** 2013-09-19

**Authors:** Elena Woods, Lili Gu, Ryan McCormick, William W Hall, Virginie W Gautier

**Affiliations:** 1 UCD-Centre for Research in Infectious Diseases, SMMS, University College Dublin, Dublin, Ireland

## Background

HIV-1 encodes an essential regulatory protein, Tat which orchestrates viral gene expression from the HIV-1 promoter. Tat activity depends on Tat hijacking the nuclear transport system to gain access to the transcriptional machinery. The sole import factor identified for Tat to date, Importin β, directly interacts with Tat-NLS to mediate its active nuclear import. While Tat localises in the nucleoli & nucleoplasm of transformed cells, in HIV-1 infected primary T-cells, Tat accumulates at the plasma membrane, where it is secreted. The different subcellular accumulation of Tat could be due to selective enhancement of the nuclear import machinery in transformed cells or alternatively, an increase in Tat nuclear export or its specific cytosolic retention in primary T cells. Of note, Tat does not contain a classical NES and its nuclear export remains to be established. Here, we characterise the bidirectional transport receptor, Importin 13 (IPO13) as a novel transport receptor mediating the nucleo-cytoplasmic shuttling of Tat.

## Methods

Co-localisation studies were performed using immunofluorescence and confocal microscopy, followed by deconvolution. For protein interaction studies, *in vitro* GST pulldowns were employed using recombinant GST-YFP-Tat and corresponding mutants with 6xhis-IPO13. *In vitro* nuclear transport assays were performed using digitonin permeabilised HeLa cells. Tat activity was monitored using HeLa-LTR-Luc reporter cells. SiRNA knockdown of IPO13 was validated by Western blot.

## Results

GST pulldowns demonstrated that Tat interacts directly with IPO13 via its NLS, as well as via additional NLS independent binding sites. *In vitro* transport assays revealed IPO13 as a novel import factor for HIV-1 Tat and identified residues 49R50K51K within Tat-NLS as essential for import by IPO13. Of note, IPO13-mediated Tat nuclear import unusually resulted in Tat nucleoplasmic distribution with weak nucleolar staining, while IPO13 import of the Tat-NLS alone resulted in a typical strong nucleolar accumulation. Importantly, IPO13 could mediate the active, CRM-1 independent nuclear export of Tat, but not Tat-NLS alone, via the Nuclear Pore Complexes. Collectively, these results suggest that the Tat-NLS is sufficient for interaction with IPO13 and nuclear import, but Tat nuclear export by IPO13 requires additional domains. Interestingly, overexpression of IPO13 induced Tat redistribution to the cytoplasm *in vivo.* Finally, siRNA mediated knockdown of IPO13 in HeLa-LTR-Luc reporter cells inhibited Tat transactivation by 60%, compared to non-target siRNA controls, highlighting the functional significance of IPO13 for Tat activity.

## Conclusions

We have identified IPO13 as an essential new transport factor for Tat and provided the first evidence of Tat nucleo-cytoplasmic shuttling, which could have functional implications for Tat cytoplasmic activities and its secretion by infected cells.

